# Dietary Supplementation of Probiotic *Lactobacillus acidophilus* Modulates Cholesterol Levels, Immune Response, and Productive Performance of Laying Hens

**DOI:** 10.3390/ani10091588

**Published:** 2020-09-06

**Authors:** Abdulaziz A. Alaqil, Ahmed O. Abbas, Hossam S. El-Beltagi, Hanaa. K. Abd El-Atty, Gamal M. K. Mehaisen, Eman S. Moustafa

**Affiliations:** 1 Department of Animal and Fish Production, College of Agricultural and Food Sciences, King Faisal University, P.O. Box 420, Al-Ahsa 31982, Saudi Arabia; aalaqil@kfu.edu.sa (A.A.A.); aabbas@kfu.edu.sa (A.O.A.); 2 Department of Animal Production, Faculty of Agriculture, Cairo University, Gamma St., Giza 12613, Egypt; 3 Department of Agricultural Biotechnology, College of Agricultural and Food Sciences, King Faisal University, P.O. Box 420, Al-Ahsa 31982, Saudi Arabia; helbeltagi@kfu.edu.sa; 4 Department of Biochemistry, Faculty of Agriculture, Cairo University, Gamma St., Giza 12613, Egypt; 5 Department of Poultry Breeding, Animal Production Research Institute, Agricultural Research Center, Dokki, Giza 12611, Egypt; Hanaa.amin@arc.sci.eg

**Keywords:** *Lactobacillus acidophilus*, cholesterol contents, immunological parameters, laying performance, chickens

## Abstract

**Simple Summary:**

Chicken eggs provide a considerable source of high-quality nutrients for human food and health. However, egg consumption may harm some people for its high contents of cholesterol and association with cardiovascular disease risk. Therefore, we investigated the possible effect of using *Lactobacillus acidophilus* (LA) as a probiotic additive in the laying hens’ diets on lowering egg-yolk cholesterol and also evaluated the immune responses and the productive performance of laying hens. The obtained results display the ability of LA, when supplemented in hen diets, to decrease the plasma triglycerides and cholesterol levels and the liver and egg yolk cholesterols in the hen. The beneficial effects of LA were also explored on some important humoral and cell-mediated immune responses in the laying hens. These positive effects led to an improvement in the productive performance of laying hens. Therefore, dietary LA supplementation could be recommended as a nutritional strategy for commercial lower-cholesterol egg production in addition to positive impacts on the performance and health of laying hens.

**Abstract:**

This study examines the effect of dietary supplementation with *Lactobacillus acidophilus* (LA) on the cholesterol levels, immune response, and productive performance of laying hens. A total of 216, 40-week-old, commercial Hy-Line brown chicken layers were randomly assigned into four treatment groups (18 birds × three replicates per group) and fed diet supplemented with 0 (control), 1 × 10^9^, 21 × 10^9^, and 31 × 10^9^ colony forming units (CFUs) of *Lactobacillus acidophilus* (LA) per kg of feed for six consecutive weeks. Results show that plasma triglycerides, low-density lipoprotein (LDL) and total cholesterols became lesser, while high-density lipoprotein (HDL) cholesterol became higher in LA-supplemented groups compared to the control. In addition, a significant reduction occurred in the liver and egg yolk cholesterol by LA supplementation. Moreover, the immunological parameters including antibody titer against sheep red blood cells (SRBCs), phytohemagglutinin (PHA)-wattle swelling test, and T- & B-lymphocyte proliferation were enhanced in laying hens supplemented with LA compared to the control hens. While the heterophil to lymphocyte (H/L) ratio decreased with LA supplementation, indicating low stress conditions in the treated hens. These positive effects for LA were further reflected on the productive performance of laying hens and improved egg production, egg weight, egg mass, and feed efficiency. Our findings indicate that LA probiotic could be recommended in laying hens’ diets for lowering egg yolk cholesterol with positive impacts on health and performance.

## 1. Introduction

Chicken eggs are considered as an important and inexpensive source of high-quality protein. In particular, eggs contain various fatty acids, vitamins, and minerals that the human body requires to be healthy [1]. However, eggs are considered a major source of dietary cholesterol, since one egg contains more than 200 mg cholesterol. Therefore, egg consumption may lead to a significant elevation in plasma cholesterol levels and may increase the risk of cardiovascular diseases (CVD) [2], which is a leading cause of people deaths worldwide, especially in low- and middle-income countries, according to reports of the World Health Organization [3]. To this end, lowering cholesterol content of eggs by genetic selection or nutritional strategies in laying hens has been a target of great research in the last few decades; however, results were inconsistent with wide variation responses [4].

Probiotics are a culture of live microorganisms that have beneficial effects on animal and poultry health when applied to their feeds [5]. Probiotics have been previously studied for their cholesterol-lowering effects in poultry [6,7]. In addition, dietary probiotics supplementation has been reported to improve growth performance, nutrient digestibility, cecal microflora balance, plasma immunoglobulins, and immunity of chickens [8]. *Lactobacillus acidophilus* is a species of non-spore-bearing lactic acid bacteria and has been used as probiotic to improve broiler [9,10] and layer [11,12,13] chicken performance.

The impact of *Lactobacillus acidophilus* (LA) on laying hen’s performance and egg cholesterol is not well-understood. It was reported that daily feed consumption, egg production, egg size, egg quality, and nitrogen/calcium retentions were improved by the addition of viable *Lactobacillus acidophilus* to the basal diets of laying hens [14,15,16], while other studies [11,12,13,17] did not observe any improvement in performance, egg weight, feed intake, feed efficiency, and livability when laying pullets were fed derived products of *Lactobacillus* culture. On the other hand, the immunomodulatory activities of probiotic bacteria have been well documented in mammals [18]. It has been shown that *Lactobacillus* culture possess beneficial effects on immune response and cytokines in chickens [17,19]; moreover, it can limit *Salmonella* colonization [20,21]. Although *Lactobacillus* is a predominant genus of bacteria normally inhabitant in poultry intestines [22,23]; however, to the best of our knowledge, there is little information available on chicken immune responses to probiotic bacteria, especially when LA is used as a probiotic. Therefore, this study aimed to evaluate the impact of dietary probiotic LA supplementation on cholesterol levels, immune response, and productive performance of laying hens.

## 2. Materials and Methods

The care and handling of the laying hens were consistent with the guidelines set by the Institutional Animal Care and Use Committee at Cairo University (CU-IACUC). If it was not mentioned otherwise, all chemical reagents and compounds used in this experiment were obtained from Sigma-Aldrich Chemical Co., St. Louis, MO, USA.

### 2.1. Lactobacillus Culture Preparation

A freeze-dried lyophilized culture of LA was obtained from a commercial company for starter cultures and media (Wisby Gmbh Co. & Kg, Niebüll, Germany). The bacteria were inoculated into de Man, Rogosa and Sharpe (MRS ) broth and incubated for 24 h at 37 °C. After that, LA cells were isolated by centrifugation at 2000× *g* for 10 min at 4 °C, then were lyophilized at –20 °C. The lactobacillus culture (LC) was diluted with a mixture of cornstarch and skimmed milk powder to finally obtain a colony forming unit (CFU) concentration of 1 × 10^9^ cells/g, based on the original detected concentration of the colony in the MRS broth. The LC was kept at 4 °C and biweekly examined for viability and desired concentration. LC was supplemented into the diet of laying hens daily to maintain the diet with viable LA cells during the experimental period.

### 2.2. Experimental Design, Birds, and Dietary Treatments

A total of 216, 40-week-old, commercial Hy-Line brown chicken layers were used in the current work. Hens were settled in an opening house system in cages (3 hens per cage) and provided with a photoperiod regimen of 16L:8D throughout the whole period of the study. A basal diet was formulated according to the nutrient requirements suggested by the management guide of the commercial Hy-Line brown chicken layers used in the present study. The ingredient composition and calculated nutrient analysis, as well as the determined chemical analysis as described by AOAC [24], of the basal diet are presented in Table 1. Hens were randomly assigned into 4 treatment groups (3 replicates per group × 6 cages in each replicate) according to dietary supplementation with 0 (control), 1 × 10^9^, 2 × 10^9^, and 3 × 10^9^ CFU of the probiotic LA per kg of feed. These treatments continued for 6 consecutive weeks (from 41 to 46 weeks of age). The productive performance of laying hens was evaluated for each treatment group, as described below. In addition, cholesterol levels in plasma, liver and egg yolk of laying hens were assayed for all groups. Furthermore, some parameters for immune response were measured and analyzed in laying hens for each treatment group. All birds were provided with ad libitum water and feed, as well as similar environmental and hygienic conditions throughout the experimental period.

### 2.3. Layer Productive Performance

The body weight of hens at the beginning and at the end of the experiment were recorded for each treatment group. Egg number and egg weight were recorded daily per hen during the experimental period. The hen-day egg production (%) was calculated for each treatment group during the whole experimental period. Average egg weight, feed consumption, and total egg mass were calculated per hen during the whole experimental period. Feed conversion ratio was calculated on the basis of egg-mass produced per unit feed consumption during the whole experimental period. The weights of hens, eggs, and feed were recorded using an electronic weighing scale with an accuracy of 10 kg × 0.5 g (Model DT580, Atrontec Electronic Tech Co., Ltd., Jiangsu, China).

### 2.4. Plasma Triglycerides and Cholesterol Analysis

At the end of the experiment (46 weeks of age), 15 hens per treatment group were fasted for 12 h, then blood samples were obtained from the brachial vein into heparinized tubes. Plasma was separated by centrifugation at 1500× *g* for 10 min. Plasma triglycerides, total cholesterol, and high-density lipoprotein (HDL) cholesterol were assayed according to Bishop [25], using colorimetric techniques of enzymatic diagnostic kits (Triglycerides 432-40201, Cholesterol 439-17501 and HDL cholesterol 431-52501, respectively; Wako Pure Chemical Industries Ltd., Tokyo, Japan). Low-density lipoprotein (LDL) cholesterol was determined according to the formula: LDL cholesterol = Total cholesterol—HDL cholesterol—(Triglyceride/5).

For liver cholesterol analysis, 9 hens per treatment group were slaughtered at the end of the experimental duration (46 weeks of age), and the livers were immediately removed and stored at 20 °C until analysis. For egg cholesterol assay, 15 eggs per group were gathered at the end of the experiment, and the yolk was separated for further analysis. Lipids in liver and yolk samples were extracted according to methods cited by Sirri et al. [4]. In brief, 1 g of the sample was put together with 3 mL of 33% KOH and 30 mL of 95% methanol in a water bath at 65 °C for 90 min. After that, the sample was vigorously shaken for 10 min with 3 mL water plus 10 mL hexane. An internal standard 5*α*-cholestan (C-8003, Sigma) was used. Cholesterol content was determined by gas chromatography (Model GC-2010, Shimadzu, Kyoto, Japan) using a column of 30 m × 0.25 mm × 0.25 μm (Model VB-1, Valco Instruments Co., Inc., Houston, TX, USA) with 100:1 split ratio and nitrogen as a carrier gas for a column flow rate of 0.54 mL/min. Temperatures of injector, column, and detector were 275 °C, 290 °C, and 340 °C, respectively.

### 2.5. Immune Response Parameters

#### 2.5.1. Total White Blood Cell (TWBC) Count

At the end of the experimental period, TWBCs were counted in 9 blood samples per treatment group using a brilliant cresyl blue stain, as described in a previous work [26]. Briefly, 490 µL of stain was mixed with 10 µL of blood sample, and 2 drops were put on a hemocytometer slide. The average count of TWBCs for each sample was determined under light microscope at magnification of 200x.

#### 2.5.2. Heterophil to Lymphocyte (H/L) Ratio

H/L ratio was manually determined in blood samples (9 samples per treatment group) taken at the end of the experimental period, as described in a previous work [26]. In brief, blood smears (twice slides for each sample) were fixed with methyl alcohol and stained by Hema-3 (cat# 22–122,911, Fisher Scientific, Pittsburg, PA, USA). The differential counts were done for a total of 200 leukocytes per slide using light microscope at magnification of 1000x with oil immersion. After averaging the cells of 2 slides, the H/L ratios were then calculated.

#### 2.5.3. Antibody Titer

The antibody titer against sheep red blood cells (SRBCs) was used as an indicator to the humoral immune response of laying hens in the present study. After 5 weeks of starting the experiment, 9 hens from each treatment group were intravenously injected with 1 mL of 5% saline suspension. Blood samples were collected from the hens one week following the injection, then sera were separated by centrifugation at room temperature (220× *g*) and stored at−20 °C until tested. The test is performed in 96-well U-bottom plates. Serial twofold dilutions of sera samples were made in a volume of 25 μL, and 25 μL of a 2% solution of SRBCs was added to each dilution. A negative control of phosphate-buffered saline (PBS) was used in the test. The plates were kindly vortexed for few seconds then kept overnight at room temperature. The titers were read when uniformly agglutinated cells had coated the bottom of the wells. Antibody titer values were expressed as log^2^ of the reciprocal of the highest dilution exhibiting visible agglutination [27].

#### 2.5.4. PHA-Wattle Test

The swelling of chicken wattle that induced by intradermal mitogen injection of phytohemagglutinin (PHA) was used as an index of cell-mediated immunocompetence [28]. At the end of the experimental period, a marked area of the wattle (9 hens per treatment group) was injected with a solution of 0.5 mg PHA in 0.1 mL sterile PBS. At 24 h post-injection, the augmentation in wattle thickness was measured (mm) as a response to the PHA-wattle immune test.

#### 2.5.5. Peripheral Lymphocyte Proliferation

At the end of the experimental period, blood samples were collected from 9 hens per group for further assays of T- and B-lymphocyte proliferation according to methods described in a previous work [29]. In each sample, the layer of peripheral blood mononuclear cells (PBMCs) was first isolated using Histopaque-1077 separation medium, washed twice with RPMI-1640 washing medium (Invitrogen Corp., Grand Island, NY, USA) supplemented with antibiotics (100 units penicillin and 100 *μ*g streptomycin per mL), and then resuspended in 1 mL of RPMI-1640. The viable lymphocytes were determined using Trypan Blue dye and plated at 1 × 10^7^ cells/mL in triplicates using 96-well U-bottom plates. Fifty μL of either Concanavalin-A (Con-A mitogen, 50 μg/mL) or Lipopolysaccharide (LPS, 10 μg/mL) was added to selected wells to stimulate T- or B-lymphocyte cell proliferation, respectively. Other wells were supplemented only with 50 μL RPMI-1640, used as control wells (non-stimulated cells). The plates were incubated (42 °C, 5% CO_2_, 48 h), then further incubated for 4 h after the addition of 15 μL of 3-[4,5-dimethylthiazol]-2,5-diphenyltetrazolium bromide (MTT, 5 mg/mL) to each well. After that, a sodium–dodecyl–sulfate solution (100 μL, 10%, dissolved in 40 mM HCl) was added to lyse the cells with the MTT crystals in each well. Finally, the optical density at 570 nm (OD570) for the experimental and blank (control) wells were recorded using an automated enzyme-linked immunosorbent assay (ELISA, Model 550 Microplate Reader, Bio-Rad Laboratories Inc., USA). Stimulating indexes for T- and B-lymphocyte cells were calculated as the ratio of the OD570 for stimulated wells to the OD570 for non-stimulated wells.

### 2.6. Statistical Analysis

All statistical analyses were carried out using SAS/STAT^®^ 9.3 software (Copyright © 2011, SAS Institute Inc., Cary, NC, USA). Data were performed to one-way ANOVA with treatment effect using the General Linear Model (GLM) procedure. The main factor was LA supplementation levels, and the means were compared for significance by Duncan’s multiple-range tests. A *p*-value of less than 0.05 was considered significant. 

## 3. Results

### 3.1. Triglycerides and Cholesterol Analysis

The effect of dietary LA supplementation on plasma triglycerides and cholesterol levels as well as liver and egg yolk cholesterol in laying hens are shown in Table 2. Administration of LA in the diet at the levels of 2 × 10^9^ and 3 × 10^9^ CFU/kg reduced (*p < 0.05*) plasma triglycerides by 23.9 and 20.8%, respectively, compared to the control group. In addition, supplementing feeds with LA at the levels of 1 × 10^9^, 2 × 10^9^, and 3 × 10^9^ CFU/kg caused a significant (*p* < 0.05) reduction in the plasma cholesterol by 10.5, 9.7, and 9.4%, and in the plasma LDL cholesterol by 13.5, 19.5, and 21.2%, respectively, compared to the control group. In contrast, plasma HDL cholesterol in laying hens treated with 2 × 10^9^ and 3 × 10^9^ CFU/kg LA was significantly (*p* < 0.05) increased by 6.3 and 9.7%, respectively, compared to the control group. With respect to the liver and egg yolk cholesterol, results indicate that dietary LA supplementation significantly (*p* < 0.05) decreased the liver cholesterol in all treated groups by approximately 14.6–21.9% when compared to the control group, while the LA supplementation at the levels of 2 × 10^9^ and 3 × 10^9^ CFU/kg significantly (*p* < 0.05) reduced egg yolk cholesterol by 23.4 and 25.5%, respectively, compared to the control group (Table 2).

### 3.2. Immune Response Parameters

Results of TWBC count, H/L ratio, antibody titer, PHA-wattle test and peripheral lymphocyte proliferation as affected by dietary LA supplementation in laying hens are summarized in Table 3. A slight increase was observed in the count of TWBCs in the treated hens with LA, but this increase was not statistically significant when compared to the control group. The inclusion of LA in laying hen diets at the levels of 2 × 10^9^ and 3 × 10^9^ CFU/kg significantly (*p <* 0.05) decreased the H/L ratio by 28.8 and 33.3%, respectively, compared to the control group. In contrast, supplementing diets of laying hens with LA significantly (*p <* 0.05) increased the antibody titer and PHA-wattle response by at least 25% and 56%, respectively, in the treated groups, compared to the control group. Moreover, supplementing diets of laying hens with LA significantly (*p <* 0.05) increased the stimulating indexes of T-lymphocyte and B-lymphocyte proliferation by at least 38% and 71% in the treated hens, respectively, compared to the control hens.

### 3.3. Layer Productive Performance

The effect of dietary LA supplementation on the productive performance of laying hens is represented in Table 4. Results show that supplementing the diet with 3 × 10^9^ CFU/kg LA significantly (*p* < 0.05) improved the hen-day egg production by 2.2 p.p. compared to the control group. In addition, providing dietary LA at 2 × 10^9^ and 3 × 10^9^ CFU/kg significantly increased (*p* < 0.05) the average egg weight by 1.7 and 1.9 g and the total egg mass by 1.3 and 4.8%, respectively, compared to the control. In contrast, the daily feed consumption of laying hens was significantly (*p < 0.05*) reduced by 3.0, 3.6, and 2.1% in the treated groups with 1 × 10^9^, 2 × 10^9^, and 3 × 10^9^ CFU/kg LA, respectively, compared to the control group. Furthermore, the feed conversion ratio (kg feed/kg eggs) of treated hens with 2 × 10^9^ or 3 × 10^9^ CFU/kg LA was significantly (*p* < 0.05) improved by approximately 8.7% when compared to the control group.

## 4. Discussion

To perform the current study, a preliminary experiment was carried out on a group of laying hens to determine the dosage and the period that induce notable effects of LA on the egg productivity. It was found that 10^9^ CFU of LA was enough to see clear results on the production of hens. It was also observed that productivity of hens started to improve from the third to the sixth week of LA administration, then it was not change when continuing till the ninth week of LA administration. Therefore, 1–3 folds of 10^9^ CFU per kg feed were considered suitable for the current experiment, and taking into account the high cost of LA culture and preparation, the period of six weeks of LA administration to laying hens was considered appropriate to carry out the present study from the economic perspective, especially when these methods might be implemented by poultry breeders. 

Results of the present study showed that supplementing LA to laying hens’ diets had depressing effects on the plasma triglycerides and the cholesterol levels in the plasm, liver, and egg yolk of laying hens. Similar cholesterol-depressing effects were reported when using other types of bacilli probiotics in the diet of broiler chickens [7,30,31]. It was also found that probiotics contained LA had a hypocholesterolemic potential effect and improved lipid profile of rat and human subjects [8]. LA may provoke the process of cholesterol elimination from the liver via bile excretion into the feces [32]. It also may provoke the reverse cholesterol transport by converting excess cholesterol into bile salts as HDL cholesterol, which then transported in the blood stream and returned to the liver [33]. In addition to previous reasons, cholesterol-depressing effect of LA may be due to cholesterol assimilation into the cellular membrane and utilization for metabolism of *Lactobacillus* itself, thereby reducing cholesterol absorption in the digestive system and preventing cholesterol synthesis [34]. 

On the other hand, administration of probiotic bacteria or their products may have immunomodulatory effects [19]. In the present study, LA supplementation did not affect TWBC count in laying hens but led to a significant reduction in the H/L ratio, reflecting low stress susceptibility in treated birds [35]. This observation suggests that high levels of LA may cause a redistribution of leukocyte components in the peripheral blood towards an increment in lymphocyte populations, which in turn stimulate humoral immune response in chickens [36]. Furthermore, there is a possible correlation between H/L ratio and stress-induced suppression of humoral immune response [37]. Results of the present study and other studies are in line with these findings since treated hens with *Lactobacillus* strains expressed a significant increase in the antibody production against SRBCs (as shown in Table 3), Newcastle disease virus (NDV) [17], or salmonella infection [38]. Regarding cell-mediated response of laying hens, the PHA-wattle test and both T- and B-lymphocyte proliferation were determined in the present study, and they were significantly increased by dietary LA supplementation. These results match previous reports [39], which found positive effects for feeding lactobacilli probiotics on immune cellular proliferation in layer- and meat-type chickens. 

Indeed, the mechanisms of how LA differentially stimulates chicken humoral and cellular immune responses are sometimes conflicting and depend on several factors, such as selection, feeding regime, strain, species, age, and tissue type of poultry [39]. For example, a counteractive relationship was observed between immunological variables, such as mitogenic leukocyte response to Concanavalin A and antibody response to SRBCs, due to selection for high body weight in turkey [40]. In our point of view, LA effects on immunomodulatory response of poultry could be well interpreted by the role of lactobacilli strains in cytokines secretion from immune system cells [41,42,43]. In this regard, it is possible that low H/L ratio and high antibody production against SRBC injection in chickens is mediated by T-helper type 1 (Th1) pro-inflammatory cytokine Interferon-γ (IFN-γ) [37]. In addition, it was found that administration of LA in mice regulated both Th1 and Th2 cytokine responses, up-regulated IFN-γ expression, down-regulated interleukins (IL-4 and IL-10) expression, and induced more expression of transforming growth factor beta (TGF-β), which is known to be associated with activation of regulatory T-lymphocyte cells (Treg cells) [44,45]. Several studies showed that Lactobacilli increase the number of Treg cells, whereas those latter particularly play a vital role in the inhibition of inflammation [46,47]. In agreement with other studies on lactobacilli administration in mice [48,49] and human [50] subjects, B-lymphocyte proliferation was observed to be increased by LA treatment in the present study. The increase in B-lymphocytes is known to be responsible for the production of immunoglobulins that participate in humoral immunity [43]. Furthermore, it is worth mentioning that low levels of plasma total cholesterol and LDL cholesterol in the treated hens with LA may lead to high expression of Th1 related cytokines and support-cell-mediated immunity [51].

Data presented in the current study showed that all parameters of productive performance, such as hen-day egg production, average egg weight, feed consumption, and feed conversion ratio, were significantly improved by dietary LA supplementation in laying hens (Table 4). Similar results have also been reported in several studies conducted in hens supplemented with *Lactobacillus*-based probiotics [7,8,52]. This improvement could be attributed to the effect of lactobacilli culture on improving intestinal microbial balance, adhering to the intestinal mucosa, secreting active metabolites, and its ability to antagonize and competitively exclude some pathogenic bacteria in chickens [53,54]. Other reports ascribed this improvement to the better nutrient digestion and absorption due to the activity of enzymes derived from Lactobacilli culture [55]. Furthermore, the addition of LA reduced stress in laying hens, as indicated by lowering H/L ratio in the present study, and improved the humoral and cell-mediated response as mentioned before (Table 3). These positive events, subsequently, decrease reproductive pathologies and enhance the performance of laying hens [56,57].

## 5. Conclusions

It is concluded that the dietary supplementation with 1 × 10^9^ CFU of LA per kg of feed had some positive effects on laying hens, including a significant decrease in the plasma cholesterol, LDL cholesterol and liver cholesterol, and a significant increase in some immune parameters, such as the antibody titer against SRBCs, PHA-wattle swelling test, and T- and B-lymphocyte proliferation. However, it seems to not affect the laying performance except from the feed intake, which was significantly decreased. The dietary supplementation with higher concentrations of LA (2 × 10^9^ and 3 × 10^9^ CFU/kg) had more significant effects on the cholesterol levels of laying hens, decreasing the plasma triglycerides, total cholesterol, and LDL cholesterol, as well as decreasing the liver and egg yolk cholesterol levels, while the beneficial HDL cholesterol was increased. In addition to the positive effects on all immunological parameters in the laying hens supplemented with LA at 2 × 10^9^ and 3 × 10^9^ CFU/kg, the H/L ratio was significantly decreased, indicating low stress conditions in the treated hens. These positive effects for 2 × 10^9^ and 3 × 10^9^ CFU/kg of LA were further reflected on the productive performance of laying hens and improved egg production, egg weight, egg mass, and feed efficiency. Therefore, dietary LA supplementation could be recommended as a nutritional strategy for commercial lower-cholesterol egg production in addition to positive impacts on the performance and health of laying hens.

## Figures and Tables

**Table 1:** The ingredients and calculated nutrient analysis as well as the determined chemical analysis of the basal diet.

Ingredients	%	Calculated Nutrient Analysis	
Yellow corn	56.55	Metabolizable energy (MJ/kg)	1.26
Soybean meal (44%)	27.60	Crude protein (%)	17.47
Wheat bran	1.00	Calcium (%)	4.02
Soybean oil	3.00	Available phosphorus (%)	0.52
Bone meal	3.00	Lysine (%)	0.95
Limestone	8.00	Methionine (%)	0.42
Salt (NaCl)	0.40	Linoleic acid (%)	2.88
Vit. & min. mixture *	0.30		
DL-Methionine	0.15		
Total	100		
Chemical analysis			
Metabolizable energy (MJ/kg)	1.28		
Dry matter (%)	89.0		
Crude protein (%)	16.75		
Ether extract (%)	6.6		
Crude fiber (%)	4.7		
Total ash (%)	12.9		
Calcium (%)	4.22		
Available phosphorus (%)	0.42		

* Vitamin and mineral mixture at 0.3% of the diet supplies the following per kg of the diet: 8000 IU vitamin A; 1500 IU vitamin D; 4 mg riboflavin; 10 μg cobalamin; 15 mg vitamin E; 2 mg vitamin K; 500 mg choline; 25 mg niacin; 60 mg manganese; 50 mg zinc.

**Table 2:** Effect of dietary *Lactobacillus acidophilus* supplementation on plasma triglycerides and cholesterol levels in plasma, liver, and egg yolk of laying hens.

Trait	*L. acidophilus* Levels (CFU/kg)			
	0	1 × 10^9^	2 × 10^9^	3 × 10^9^
Plasma triglyceride, mg/dl	210.1 ± 7.01 ^a^	198.5 ± 7.12 ^a^	159.9 ± 5.98 ^b^	166.4 ± 6.03 ^b^
Plasma Cholesterol, mg/dl	136.6 ± 4.54 ^a^	122.3 ± 4.04 ^b^	123.3 ± 3.77 ^b^	123.7 ± 3.28 ^b^
HDL cholesterol, mg/dl	61. 8 ± 1.91 ^b^	60.0 ± 1.85 ^b^	65.7 ± 1.84 ^a^	67.8 ± 1.64 ^a^
LDL cholesterol, mg/dl	70.3 ± 1.80 ^a^	60.8 ± 1.41 ^b^	56.6 ± 1.64 ^c^	55.4 ± 1.45 ^c^
Liver cholesterol, mg/g	4.1 ± 0.24 ^a^	3.5 ± 0.21 ^b^	3.3 ± 0.20 ^b^	3.2 ± 0.24 ^b^
Yolk cholesterol, mg/g	13.7 ± 1.08 ^a^	11.4 ± 1.05 ^ab^	10.5 ± 0.77 ^b^	10.2 ± 0.87 ^b^

Data are represented as mean ± standard error (n = 9 for the liver cholesterol parameter; n = 15 for the other parameters). ^a, b and c^ Means within the same row with different letters are significantly differed (*p* < 0.05). CFU, colony forming unit; HDL, high-density lipoprotein; and LDL, low-density lipoprotein.

**Table 3:** Effect of dietary *Lactobacillus acidophilus* supplementation on immune response parameters of laying hens.

Trait	*L. acidophilus* Levels (CFU/kg)			
	0	1 × 10^9^	2 × 10^9^	3 × 10^9^
TWBC count (10^3^/mm^3^)	43.96 ± 6.184	47.72 ± 5.902	50.78 ± 5.132	52.88 ± 5.877
H/L ratio	0.66 ± 0.067 ^a^	0.54 ± 0.077 ^ab^	0.47 ± 0.084 ^b^	0.44 ± 0.087 ^b^
Antibody titer (Log^2^)	6.37 ± 0.484 ^b^	7.99 ± 0.644 ^a^	8.62 ± 0.738 ^a^	8.48 ± 0.822 ^a^
PHA-wattle test (mm)	0.32 ± 0.030 ^b^	0.50 ± 0.055 ^a^	0.54 ± 0.064 ^a^	0.57 ± 0.050 ^a^
T-lymphocyte proliferation *	3.47 ± 0.399 ^b^	4.80 ± 0.424 ^a^	5.01 ± 0.360 ^a^	5.40 ± 0.564 ^a^
B-lymphocyte proliferation *	2.29 ± 0.331 ^b^	3.92 ± 0.349 ^a^	4.16 ± 0.238 ^a^	3.96 ± 0.231 ^a^

Data are represented as mean ± standard error (n = 9). ^a, b^ Means within the same row with different letters are significantly differed (*p* < 0.05). * T- and B-lymphocyte proliferation were expressed as the mean of stimulating indexes calculated per sample for each parameter. CFU, colony forming unit; TWBC, total white blood cells; H/L, heterophil/lymphocyte; and PHA, phytohemagglutinin.

**Table 4:** Effect of dietary *Lactobacillus acidophilus* supplementation on productive performance of laying hens.

Trait	*L. acidophilus* Levels (CFU/kg)			
	0	1 × 10^9^	2 × 10^9^	3 × 10^9^
Initial body weight (g)	1873.9 ± 1.49	1873.3 ± 1.21	1872.8 ± 1.16	1872.5 ± 1.29
Final body weight (g)	1889.2 ± 1.09	1887.8 ± 1.35	1890.0 ± 1.81	1887.2 ± 1.47
Egg production (%)	93.2 ± 0.54 ^b^	93.6 ± 0.61 ^b^	93.9 ± 0.42 ^b^	95.4 ± 0.38 ^a^
Egg weight (g)	61.7 ± 0.41 ^b^	61.8 ± 0.40 ^b^	63.4 ± 0.39 ^a^	63.6 ± 0.34 ^a^
Egg mass (g)	2419.5 ± 25.42 ^b^	2428.3 ± 31.24 ^b^	2488.7 ± 23.48 ^a^	2536.8 ± 31.65 ^a^
Feed Intake (g/d)	131.3 ± 2.44 ^a^	127.4 ± 2.14 ^b^	126.6 ± 2.39 ^b^	128.5 ± 2.33 ^b^
Feed conversion ratio (kg feed/kg eggs)	2.3 ± 0.07 ^a^	2.2 ± 0.10 ^ab^	2.1 ± 0.07 ^b^	2.1 ± 0.10 ^b^

Data are represented as mean ± standard error (*n* = 18). ^a,b^ Means within the same row with different letters are significantly different (*p* < 0.05).
